# Physician-Modified Endovascular Grafts for Zone-2 Thoracic Endovascular Aortic Repair

**DOI:** 10.1055/s-0042-1742696

**Published:** 2022-05-31

**Authors:** André B. Queiroz, Jackson B. Lopes, Vanessa P. Santos, Pedro B. A. F. Cruz, Ronald J. R. Fidelis, José S. Araújo Filho, Luiz C. S. Passos

**Affiliations:** 1 Centro de Doenças da Aorta - CDA, Division of Vascular and Endovascular Surgery, Cardiac Surgery, Cardiology and Anesthesia, Universidade Federal da Bahia, Hospital Ana Nery, Salvador-Bahia, Brazil; 2 Division of Vascular Surgery, Universidade Federal da Bahia, Hospital Universitário Professor Edgar Santos, Salvador-Bahia, Brazil

**Keywords:** aortic arch, endovascular, TEVAR, fenestration, physician-modified endograft

## Abstract

**Objective** This study aims to describe our technique and early experience with physician-modified endovascular grafts (PMEGs) for aortic arch diseases in zone 2. We used a total endovascular technique based on a single fenestrated endograft to preserve left subclavian artery (LSA) patency.

**Methods** From December 2019 to August 2020, six consecutive patients with a variety of thoracic aortic diseases were treated with handmade fenestrated thoracic aortic grafts: four aortic dissections, one penetrating aortic ulcer, and one intramural hematoma. The planning, endograft modification, surgical technique, and follow-up of the patients were described. We evaluated immediate technical success and after 30 days, the LSA patency, Type-1 endoleak, and postoperative complications.

**Results** Thoracic endovascular aortic repair (TEVAR) was performed for zone 2 in all cases. Immediate technical success, defined as successful alignment of the LSA with a covered stent and no Type-1 endoleak, was achieved in all cases. Patients had a 30-day follow-up computed tomography, which demonstrated LSA patency and no Type-I endoleaks. To date, no strokes, left arm ischemia, paraplegia, or conversions to open surgery have been reported; one patient operated for acute Type B dissection died during the early follow-up.

**Conclusion** TEVAR for zone 2 with a PMEG to maintain LSA patency achieved technical success and early durability. It is expected that with longer follow-up and a larger number of cases, these results will be confirmed.

## Introduction

Since the first implantation by Volodos in 1987, thoracic endovascular aortic repair (TEVAR) has not just become an alternative but has been proven safe and effective, replacing open surgery as the therapy of choice for a variety of thoracic aortic pathologies. 1 Left subclavian artery (LSA) coverage may be necessary in up to 40% of TEVAR cases to achieve an adequate seal zone. 2 However, LSA occlusion may be related to a higher risk of downstream ischemic complications, such as spinal cord ischemia, stroke, and left arm ischemia. 2 3 4

A hybrid technique with surgical revascularization of the LSA has been the main option for managing these patients in recent years. 5 However, revascularization may be related to morbidities such as nerve injury, lymphatic leakage, graft infection, or stroke. 6 7 8

Total endovascular approaches have improved over the years from options, such as the chimney technique and in situ fenestrations, to more sophisticated endovascular solutions, such as branched or fenestrated repair. 9 10 However, branched or fenestrated devices are not yet widely available for this region. Widespread dissemination has been hampered by regulatory issues and the cost and time required to manufacture the device.

Some of these issues may be overcome by modifying available endovascular grafts. The term physician-modified endovascular graft (PMEG) was first used by Starnes in 2012, 11 and other authors have described encouraging initial experiences with thoracic endovascular graft modifications for zone 2. 12 13 Herein, we present our initial experience with PMEG and early outcomes for a variety of aortic arch diseases in zone 2, using a single fenestrated endograft to preserve subclavian artery patency.

## Materials and Methods

This series included six consecutive patients with various aortic pathologies who underwent zone-2 TEVAR, using PMEG with a single fenestration to preserve LSA. Inclusion criteria for the modified devices were as follows: preprocedural diagnosis of thoracic aortic diseases with a neck length <20 mm from the LSA and ≥20 mm from the left common carotid artery. A minimal distance of 8 mm between the left common carotid artery and the LSA was necessary. All procedures were performed at the Ana Nery Hospital of the Federal University of Bahia, Brazil. After thoroughly informing all patients and their families about the off-label use of the graft and its risks and benefits, they granted written informed consent.

### Planning and Sizing

We used Horos version 3.3.6 (Horos Project, Annapolis, MD), a dedicated medical image viewer, to plan the procedures. Centerline luminal reconstruction was performed, and the distance between the left common carotid artery and the LSA were measured in the outer curvature of the arch. Three-dimensional volume rendering reconstruction was used to determine the best angle for arch visualization during the procedure.

Since the aim was to preserve LSA flow, a single fenestration was planned as previously described by Zhu et al. 13 Oversizing ranging from 10 to 20% was planned for all cases.

### Device Modification

**Fig. 1:**
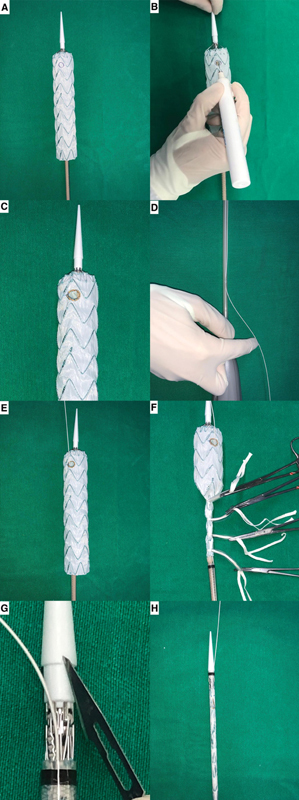
Sequential images show the endograft fully unsheathed on the back table and the fenestration site marked with a sterile pen ( **A** ). The fenestration for the left subclavian artery was made using thermal cautery ( **B** ). The edge of the fenestration was reinforced using a radiopaque wire ( **C** ). A 0.035 guidewire was passed through the sheath ( **D** ) and exited the endograft through the fenestration ( **E** ). The endograft was resheathed using umbilical tapes ( **F** ) and a small groove was made in the tip of the introducer sheath ( **G** ) to better accommodate the guidewire ( **H** ).

All procedures were performed under general anesthesia. The endograft was modified during anesthetic induction and common femoral dissection. The devices (Valiant Captivia, Medtronic, Santa Rosa, CA) were unsheathed under sterile conditions on the back table, without releasing the free-flow tip capture mechanism. A sterile marking pen was used to mark the positions and a ruler was used to measure the fenestrations. An 8-mm circular fenestration was made for the LSA using thermal cautery. This fenestration was reinforced circumferentially with a 0.035-radiopaque wire (Anaconda, Vascutek, Scotland, United Kingdom). Radiopaque marks were also used to mark the opposite side of the endograft ( Fig. 1 ).

A hole was made in the device's introducer sheath using a 21G needle. A 0.035 cm × 260 cm guidewire (Road Runner, Cook Inc., Bloomington, IN) was passed through the needle hole until it exited the sheath's extremity. It was then inserted into the endograft and advanced through the fenestration. The endograft was resheathed with umbilical tape and the preloaded guidewire emerged from the sheath under the tapered tip of the introducer, where we made a small groove with a number 11 blade to better accommodate the preloaded guidewire.

### Surgical Technique and Device Implantation

The common femoral artery (CFA) and the left brachial artery (LBA) were exposed by surgical incision, and the opposite CFA was accessed by puncture. Constant dialogue was established with the anesthesia team, especially during endograft deployment to ensure low blood pressure.

A guidewire was passed through the right CFA and exteriorized using a snare (One Snare, Merit Medical, Jordan, UT) in the left upper limb to obtain a through-and-through wire. An over-the-wire angioplasty balloon (Passeo-35, Biotronik, Bulach, Switzerland) was introduced into the LBA access using the through-and-through wire, emerging from the CFA. This balloon catheter was chosen for its length (130 cm), since it is longer than diagnostic catheters. Using the same femoral introducer, an extra-stiff Lunderquist 0.035 cm × 260 cm (Cook Inc., Bloomington, IN) was positioned in the ascending aorta.

The PMEG was visualized under radioscopy to confirm the positioning of the radiopaque marks and was then introduced over the Lunderquist wire until close to the femoral artery. The preloaded wire was introduced trough the tip of the balloon catheter until it exited in the left arm. The endograft was gently introduced up to the descending thoracic aorta while the balloon catheter was retracted. An aortogram was obtained with a pigtail catheter from other CFA. The graft was advanced up to the ascending aorta and positioned exactly at the intended position. At this point, systolic blood pressure was reduced and maintained under 70 mm Hg.

**Fig. 2:**
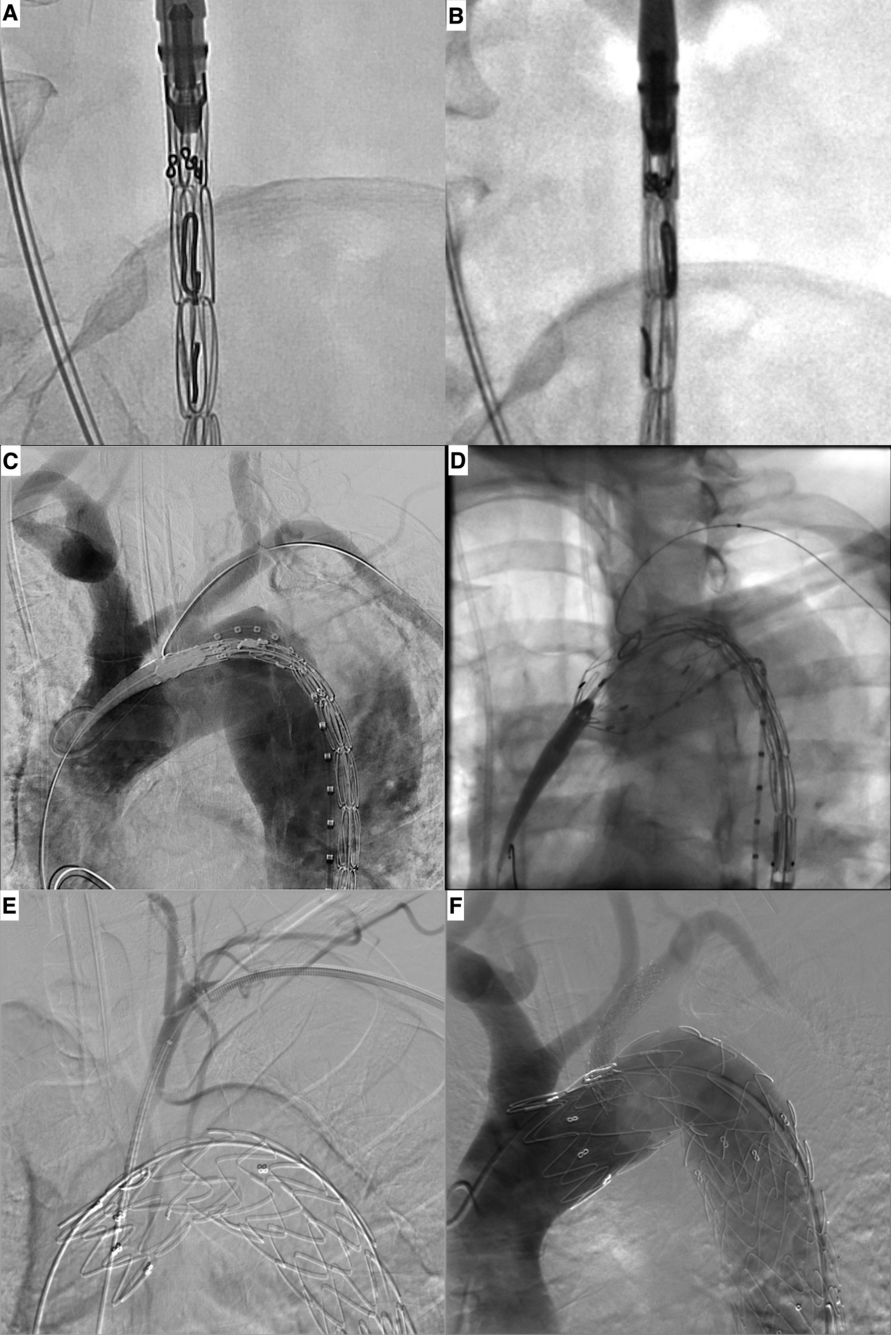
Intraoperative images demonstrate the radiopaque marks in the resheathed endograft in anterior ( **A** ) and lateral ( **B** ) views. Aortography demonstrates the aortic arch anatomy ( **C** ). The endograft partially unsheathed and placement of an angioplasty balloon trough the fenestration ( **D** ). Angiography demonstrates the vertebral artery ( **E** ) and a completion aortography shows the endograft positioning, the covered stent patency, and no endoleak ( **F** ).

The proximal part of the graft was deployed and an 8-mm angioplasty balloon (Passeo-35, Biotronik, Bulach, Switzerland) was advanced from the LBA through the fenestration using the preloaded wire. The balloon was inflated to maintain the graft position and the graft was fully deployed. The balloon was deflated, while a 7- or 8-Fr 90-cm sheath (Flexor, Cook Inc., Bloomington, IN) was advanced through the fenestration from the CFA, and a Lifestream covered stent (Bard, Tempe, AZ) was deployed 5- to 10-mm inside the endograft, aligning the hole. The positioning must be accurate to avoid occluding the left vertebral artery. The proximal portion of the covered stent was flared using a 12-mm standard angioplasty balloon. Finally, the angiography was completed, demonstrating the patency of the supra-aortic branches and the TEVAR results ( Fig. 2 ). If any proximal endoleak was seen, a compliant balloon was used to better accommodate the endograft, while the 12-mm balloon was reinflated to maintain the covered stent structure.

### Follow-up

**Fig. 3:**
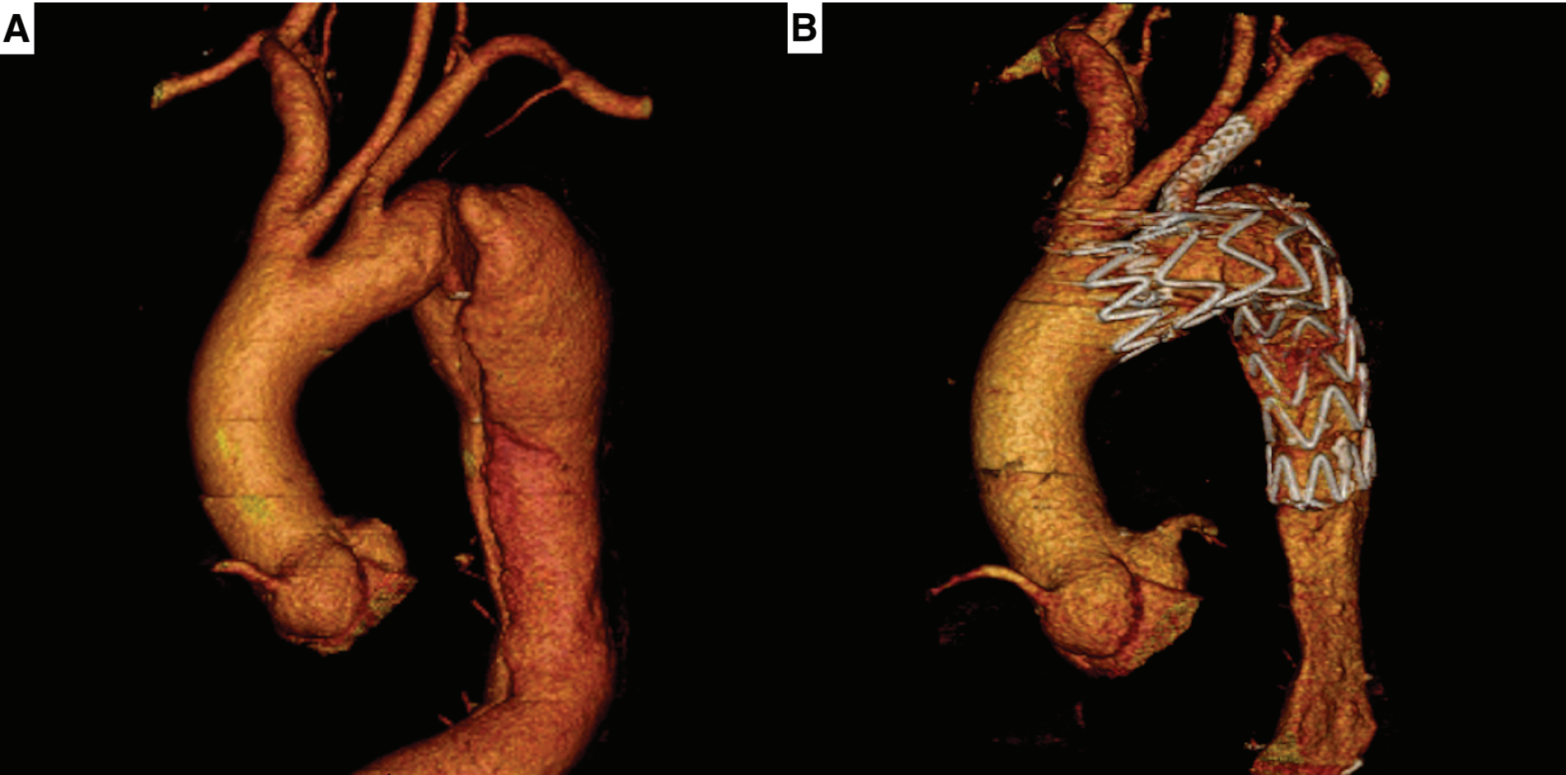
Three-dimensional computed tomography reconstructions show the preoperative image of a Type B aortic dissection beginning close to the left subclavian artery ( **A** ), and the postoperative image with the physician-modified endograft well positioned in the distal arch, with patency of left subclavian artery, and no endoleak ( **B** ).

Postoperative follow-up included routine visits to the outpatient department, and contrast-enhanced computed tomography was scheduled after 30 days ( Fig. 3 ). Early outcomes included immediate technical success and 30-day LSA patency, endoleaks, and postoperative complications. Technical success was defined as successful implantation of the fenestrated endograft into the thoracic aorta with appropriate covered stent positioning through the fenestration into the LSA and no evidence of Type-1A endoleak.

## Results

### Patient Demographics

From November 2019 to August 2020, six patients (two women: 33%) underwent TEVAR with PMEG using a single fenestration to preserve LSA patency. The mean age was 64.8 years (range: 57–75 years). In this series, two operations were elective and four were urgent repairs. The elective repairs included one patient with a right aberrant subclavian artery who underwent a right subclavian-carotid bypass to avoid a bilateral bypass and possible nerve injury. There were two chronic, one subacute, and one acute Type-B aortic dissections, one penetrating aortic ulcer, and one intramural hematoma. All dissections had surgical indication related to large diameters (>55 mm). The patient operated on in the acute phase had refractory pain despite clinical treatment.

### Endovascular Graft Configuration

The device used in all cases was a Valiant Captivia thoracic stent graft (Medtronic Inc., Santa Rosa, CA). Three cases required two thoracic endografts, while the others required only one. The diameter of all fenestrations was 8 mm, and the mean LSA diameter was 9 mm (range: 8.8–9.5 mm). The mean diameter of the covered stents was 9.8 mm (range: 9–10 mm).

The median time it took to modify the devices was 54 minutes (range: 40–82 minutes). The mean neck length from the left common carotid artery to the proximal portion of the lesion was 28 mm (range: 21–40 mm).

### Perioperative Data and Outcomes

The CFA was accessed to introduce the PMEG in five patients, and a cut-down left common iliac conduit was used in one female patient due to small external iliac arteries. Technical success was achieved in all cases, including successful placement of the PMEG into the thoracic aorta with adequate covered stent positioning through the fenestration into the LSA and no Type-1 endoleak. One case of LBA disruption occurred during manipulation due to a small arterial diameter. An end-to-end reconstruction was immediately performed with good results.

At 30-day follow-up, five patients were alive. Contrast-enhanced computed tomography was performed in the five cases which showed LSA patency and no Type 1 endoleaks. One patient, who was operated on for acute dilated Type B aortic dissection and refractory pain, presented sudden death on the second day after surgery. Echocardiography showed no retrograde dissection, and we had no opportunity to perform a computed tomography or a cardiac catheterization. The cause of death was unclear, and we could not rule out other causes related to the aorta.

There have been no reports of left arm ischemia, paraplegia, conversion to open surgery, or secondary open procedures. No patients required surgical LSA revascularization.

## Discussion

The Society for Vascular Surgery Committee on Aortic Disease began recommending routine LSA revascularization in 2009. 5 This approach has been extensively debated in the literature with respect to distal aortic arch diseases, since it often requires multiple surgical interventions and longer operative times. Some arguments in favor of this approach are the prevention of stroke, spinal cord ischemia, and left arm ischemia. 4 6 14 Nevertheless, some groups still use a selective approach, occluding a varying percentage of LSA in their series, 3 5 15 especially those performed in urgent situations.

In a retrospective analysis, Delafontaine et al 3 found lower pulmonary and neurological complications for endovascular LSA revascularization than the conventional open technique. Thus, this complete endovascular solution for the treatment of aortic arch diseases in zone 2 represents important progress for aortic surgery.

Chimney and in situ laser fenestration techniques have been used with reasonable results. 9 16 17 Hogendoorn et al 16 evaluated the chimney technique in different aortic arch pathologies and found a variable occurrence of endoleaks. Parallel grafts can involve gutters, which may cause Type 1A endoleaks, a constant concern when using this technique. Therefore, longer sealing zones are required to achieve better graft apposition. Another related concern is the patency of the covered stent due to possible compression by the endografts, which may require angioplasty with bare stents, thus requiring attention during follow-up. 9

Off-the-shelf branched devices have already been tested in selected studies but are not yet widely available. 18 19 Recently, some authors have described the use of PMEG with a single fenestration to preserve LSA patency, employing similar approaches. 12 13 This case series represents our initial experience with PMEG to treat zone-2 aortic arch diseases. There was a 100% immediate technical success rate, which was maintained in early follow-up, with LSA patency in all cases and no Type-1A endoleaks. There were no strokes, left arm ischemia, or paraplegia.

Stroke remains one of the major concerns in aortic arch endovascular repair. In the majority of cases, cerebral events are related to atheromatous embolisms in calcified aortic arches. Arch manipulation can be minimized by using preloaded wires, lower profile devices, adequate patient selection criteria, and rigorous planning. Such factors can reduce the incidence of these potentially disastrous complications. Wires and graft manipulation in the aortic arch and the potential for air embolism are significant technical factors related to the procedure. The graft sheath should always be irrigated abundantly with saline solution. Some authors have advocated the use of carbon dioxide before saline infusion to reduce the amount of air captured in the endograft. 20

The technique described here is based on the use of a single fenestration for the LSA. Thus, as a general concept for fenestrations, we only used this approach when the LSA originated from a nondilated aorta. This approach should be avoided in aneurysms involving the LSA origin; branched techniques should achieve better results in these patients.

Tortuosity is intrinsic to the aortic arch, so the precise positioning of the graft in this area is challenging. To overcome this issue, we based our technique on rigorous planning and certain mechanisms to achieve better accuracy, such as radiopaque marks to enhance visualization of the graft position, and a preloaded guidewire. Preprocedural computed tomography can predict the majority of traps that might occur during surgery, including problems with access vessels, descending aorta tortuosity, true and false lumen identification, and choosing the best view of the arch for the most suitable sealing zone. 21 Suturing a radiopaque wire circumferentially to the fenestration edge is important to enable a better connection between the fenestration and the covered stent. It is also crucial for identifying the position of the PMEG in the aortic arch, since the movements made in the device's grip are not regularly transmitted to its distal extremity. A radiopaque mark on the opposite side of the fenestration is another valuable modification that could help prevent rotational misalignment.

PMEG durability is a matter of concern for every group that works with these devices. In a systematic review, Georgiadis et al 22 compared the use of PMEG and off-the-shelf devices in the thoracoabdominal region of 308 patients (936 target vessels) and found no significant adverse events, as well as similar safety and effectiveness in both groups. Other authors working with PMEGs for the aortic arch demonstrated durable and safe results in the midterm follow-up. 23 However, careful long-term follow-up is required due to potential late complications involving durability and migration of the main endograft and the covered stent. 24

### Limitations

The technique presented in this initial study has certain limitations regarding aortic, subclavian, and access vessel anatomy. Aortic aneurysms or subclavian dilations involving the LSA origin preclude the use of this technique due to potentially poor apposition between the LSA and the fenestration. Aortic and iliac vessel tortuosity and narrowing can also involve potential issues. Branched endografts and lower profile devices are required to address these conditions. We believe that more complex cases involving zones 0 and 1 should be managed with similar techniques, that is, one fenestration combined with cervical debranching or more fenestrations for total endovascular repair.

## Conclusions

PMEG with a single fenestration is a feasible option for treating distal arch diseases that require sealing in zone 2. This therapeutic approach is valuable because it preserves LSA patency, reducing the number of procedures and the risks related to open revascularization. Although the number of patients in our sample is small and the long-term durability of this procedure is still unknown, our results encourage the use of this endovascular approach, and we hope that advances in endovascular surgery will lead to off-the-shelf devices suitable for most cases. Shifting to a single endovascular procedure to maintain LSA patency should lead to more liberal LSA preservation with total endovascular techniques.
